# Exploring the experiences of people living with dementia in Dementia Friendly Communities (DFCs) in Northern Ireland: a realist evaluation protocol

**DOI:** 10.1186/s12877-023-04090-y

**Published:** 2023-06-09

**Authors:** Stephanie Craig, Gary Mitchell, Peter O’ Halloran, Patrick Stark, Christine Brown Wilson

**Affiliations:** School of Nursing and Midwifery, Queen’s University Belfast, Belfast, Northern Ireland

**Keywords:** Dementia friendly communities, Dementia friendly initiatives, Social participation, Realist review, Caregivers, Dementia

## Abstract

**Background:**

The aim of this study is to 1) explore the experiences of people living with dementia interacting with DFCs and 2) identify factors that influence empower and support people living with dementia to live successfully in DFCs. The main elements of a DFC are related to people; communities; organisations and partnerships. There are over 200 organisations recognised as dementia-friendly in Northern Ireland (NI). This realist evaluation is to understand how DFCs work for people living with dementia, how positive outcomes are achieved, for whom and in what contexts do DFCs work best.

**Methods:**

A realist evaluation using case study methodology. The process evaluation includes a realist review of the literature, non-participant observation of people living with dementia in their local communities, semi-structured interviews to explore the facilitators and barriers to living well in DFCs and focus groups comprised of people living with dementia, family caregivers and people working in DFCs to support Context- Mechanisms and Outcomes (CMOs). This four-stage realist assessment cycle is used, including iterative rounds of theory development, data gathering, and theory testing. In the end, analysis will reveal context mechanisms that influence how dementia-friendly communities operate and present an initial theory of how people think, which, if adopted, may be able to alter current contexts so that "key" mechanisms are activated to generate desired outcomes.

**Discussion:**

To give confidence in moving from hypothetical constructions about how DFCs could function to explanations of possible or observable causal mechanisms, the realist evaluation of a complex intervention incorporates a variety of evidence and perspectives. Despite playing a significant role in a person with dementia's everyday life, it appears that little is known about how communities function to achieve intended results. Even though there has been a lot of work to pinpoint the fundamentals and crucial phases of building DFCs, it is still unclear how people living with dementia benefit the most from these communities. This study intends to advance our understanding of how outcomes are produced for people living with dementia by contributing to the underlying theory of DFCs as well as addressing the primary research objectives.

**Supplementary Information:**

The online version contains supplementary material available at 10.1186/s12877-023-04090-y.

## Background

The World Health Organisation [1] defines dementia as a syndrome in which cognitive performance deteriorates beyond what would be expected as a part of normal biological ageing. Dementia is an umbrella term [2] that encompasses a wide spectrum of progressive neurological illnesses, with over 200 subcategories. Alzheimer's disease is the most prevalent kind of dementia [3] it is a degenerative brain ailment that can occur alone or in combination with other illnesses, which account for 60-70 percent of cases [4]. Other kinds of dementia include Vascular Dementia, Frontotemporal Dementia, and Lewy bodies Dementia [5]. While the clinical manifestations of dementia are always unique to the individual [6] short-term memory problems, communication difficulties, visual and hearing distortion, increasing functional decline, changes in personality and distress are common symptoms [7]. As a result, policymakers must make it a priority to create environments and communities that are supportive of people living with the condition [1].

Living with dementia is an important issue and one that affects whole societies all over the world. Dementia Friendly Communities (DFCs) are being developed to help people with dementia in such societies. According to Alzheimer Disease International, DFCs can be defined as a place or culture in which people with dementia and their care partners can feel empowered, supported, and included in society [8]. A dementia-friendly community (DFC) is an international initiative defined as a place or culture where individuals with dementia are recognised, valued, supported, and confident in their ability to contribute to society [9]. DFCs are key in helping people with dementia to live well and stay active members of their communities. The people involved in a DFC will understand dementia and know about ways to empower them to live well with the condition. The underpinning ethos of DFCs is that they help people with dementia to achieve their highest possible quality of life, remain empowered, understand their rights, and realise their full potential [10]. DFCs are therefore beneficial to people with dementia and their family carers' quality of life [11]. Improving dementia awareness, or what has more recently been referred to as dementia friendliness, has been considered the cornerstone to developing DFCs [12].

While there has been much global research [13–15] carried out on DFCs, there has also been some empirical work carried out locally in NI. An evaluation of DFCs in NI took place in 2017 by Corry and Leavey [16] pre-COVID-19, testing 16 different DFC models to provide a foundation for NI. Following this initial evaluation, and the emergence of COVID-19, there has been no empirical evaluation that has sought to understand how DFCs have been sustained or continued to be developed in NI. The influence of established DFCs, and emergence of new DFCs, is particularly important given the impact of the global Coronavirus pandemic on social contact within many communities and organisations [17].

The aim of this study is to 1) explore the experiences of people living with dementia interacting with DFCs and 2) identify factors that influence empower and support people living with dementia to live successfully in DFCs. The research will be conducted in NI and will consider both urban and rural groups.

## Methods/design

### Methods

This study will employ a case study design using realist evaluation [18] while using a qualitative methodology to provide light on participants' perceptions and experiences. This is a theory-driven methodology that supports researchers in evaluating social programmes, such as DFCs [19]. Realist evaluation, as opposed to a traditional cause-effect, non-contextual style of research, suits the complex social intervention of DFCs as an alternate lens to traditional empirical evaluation techniques, as this perspective seeks to determine what works, for whom, in what circumstances and to what degree [20]. Realist research employs a generative causality theory [21, 22]. In other words, the outcomes we see are caused by invisible forces and causal processes that function (or don't) depending on the context in which they take place [23]. As a result, different contexts provide different outcomes. Realist approaches acknowledge that certain programmes are more effective than others and are not always universally successful [24]. Programs that are implemented in various contexts use various mechanisms and yield various patterns of outcomes. Since this is the case, realist evaluation and synthesis do not aim to ascertain the "average effect" of a programme or provide a response to the query "what works? " Instead, they seek to explain how, why, and for whom a programme or policy is effective [25].

The goal of realist evaluation is to gain a better knowledge of how and why different initiatives and programmes perform in diverse situations, which is important to understand the complex intervention of DFCs. It is highly focused on causation determining which efforts and how they contribute to certain outcomes [26]. This method can be considered as the most appropriate when assessing new initiatives, pilots or programmes [27], or any intervention when there is evidence that the project or programme works but it is unclear how, why, or for whom, to expand, replicate or scale up the intervention. This allows for a more in-depth examination of comparisons between various groups and subgroups [28].

### Design

**Fig. 1:**
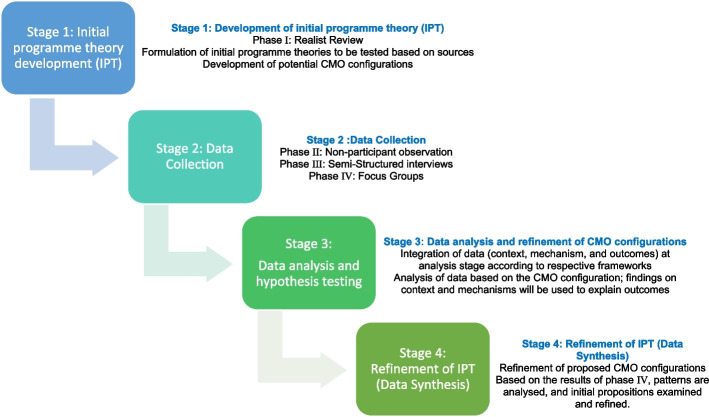
The Realist Evaluation cycle used in this study (adapted from Pawson and Tilley)

This study will be conducted in four stages (Fig. 1) as indicated further in the manuscript.

- Stage 1: Development of initial programme theory (IPT)
  - *Phase I*) a realist review of the literature to inform the development of CMO configurations and definition of cases.
    - a theory-driven approach employing context-mechanism-outcome (CMO) configurations to explain "what works, how, why, in which contexts, for whom, and to what extent [18].”
      - The heuristic known as CMO is used to explain generative causation. The relationship between a context, a mechanism, and an outcome of interest in a certain programme is helped by CMOs. CMOs might deal with the entire programme or just particular elements of it. The creation or improvement of (programme) theories is based on the configuration of CMOs [29]
- Stage 2: Data Collection (Collected in three phases)
  - *Phase II*) a qualitative exploration of the day-to-day experience of people with dementia in the DFC cases through non-participant observations.
  - *Phase III*) semi-structured interviews with people living with dementia (and their family member if appropriate) about their experiences of living in DFCs.
  - *Phase IV*) focus groups involving people living with dementia, their careers and staff working in DFCs.
- Stage 3: Data Analysis and refinement of CMO configurations
- Stage 4: Refinement of IPT (Data Synthesis)

## Establishment of expert reference group

The Expert Reference Group (ERG) will include up to 12 individuals comprised of people who live with dementia, dementia carers, dementia care clinicians, researchers in dementia and Dementia champions from DFCs across NI. The ERG will review project progress and outputs, ensure protocol adherence, and act as a network based on the project's dissemination objectives. The ERG will also offer advice on all pertinent project aspects. At three key junctures in the project's life cycle—during the project launch, before stage 2 data collection and when evaluation results are being discussed—the ERG will get together to discuss intervention development. ERG members will occasionally be consulted privately about study-related issues outside of these meetings. All communications will take place on MS Teams, which complies with The United Kingdom’s General Data Protection Regulation (UK GDPR), or over the phone.

## Study procedures

### Stage1: Phase I

#### Development of Initial Programme Theory (IPT)

To identify the evidence base, theory and potential outcomes, the team will firstly conduct a realist review (phase I) of the international literature on dementia-friendly communities using RAMESES I (Realist and Meta-narrative Evidence Syntheses: Evolving Standards) reporting standards for realist reviews [30]. This review will develop theories about how DFCs work within an international context that will inform initial CMO configurations that will guide the data collection.

### Stage 2

#### Data collection

Initial Programme Theory (IPT) will be reviewed at each stage of data collection to build new questions and develop an understanding of the next stage. Stage two of the realist evaluation cycle will focus on data collection. This will occur in three phases (phase II, phase III and phase IV).

##### Phase II

Data collection will be comprised of non-participant observation with approximately 10 people with dementia and their carers (where appropriate) when undertaking day-to-day activities within DFCs. For example, observing someone with dementia in a bank or supermarket as they complete daily tasks or using public transport which is a service identified as dementia friendly. The researcher will be observing the day to activities of the person with dementia and their carers with their knowledge that they are engaged in a research project. This approach builds on previous work where people living with dementia have been asked about their experiences as ‘mystery shoppers’ [31]. Non-participant observation is recommended as an effective way to access the experience of older people with cognitive impairment [32] and has been successfully used within a realist evaluation in this population [33].

Non-participant observation will be semi-structured and informed (but not restricted) by an observation topic guide (Supplementary file 1) developed from the findings of the realist review in consultation with the ERG [34]. All observations will take place in a public location determined by the participant. The length of observation will be a maximum of twenty minutes. This is an estimate based on ERG feedback but may vary slightly depending on activity, for example, a participant might spend one hour in a supermarket compared to twenty minutes in a café. If the participant is visiting two locations in one day, this will be two separate observations. This will enable the researcher to observe first-hand the interactions between the person living with dementia, the DFC physical environment and the service staff that speak with the person living with dementia during their experience. Non- participant observation will be recorded via field notes which will be updated in real-time as near to the events in time as possible, and these will be later transcribed. No audio will be recorded as the participant will be authentically engaging with the community around them. All names will be replaced with a code. All transcripts will be retained securely for up to 5 years after the end of the study before being destroyed.

##### Phase III

All the people living with dementia from phase II will be invited to take part in semi-structured interviews regarding their experiences during non-participant observation (one interview for each period of observation). This phase may also involve a family member if requested by the person living with dementia. During phase III, the research team will also ask the participant about their experiences in other DFCs, beyond what was observed by the researcher. A storytelling approach will enable participants to describe their experiences with the researcher [35, 36]. With the participants' consent, all semi-structured interviews will be audio-recorded. These qualitative findings will support us to refine the CMO configurations. These interviews will take place at a time and place decided by the person living with dementia.

##### Phase IV

The final method of data collection will take place after phases II and III. Phase IV will use online focus group interviews to support analysis of the findings from phases II and III. It is anticipated that 4-5 mixed focus groups comprising of people living with dementia, carers and people working in DFCs will take place (6-8 per group within each case study totalling up to 32 people). These will last around 30-40 minutes and focus on public perception. Focus groups will take place remotely via MS Teams. This will support a wide variety of stakeholders to participate in data collection. With participant's permission online focus groups will be video and audio recorded. The purpose of the focus groups will be to critically discuss data obtained from phases I-III to support the refining of the CMO configurations from the perspective of those actively engaged in the DFC.

Stage two outlines how data collection will take place through three key phases. All phases of data collection will support the realist evaluation cycle.

### Stage 3

#### Data analysis and refinement of CMO configurations

The methods used to acquire the data are centred on measuring the results, collecting information about the context and potential operating mechanisms, and determining how these relate to one another and work to produce the outcomes. Stage 3 will see the Integration of data (context, mechanism, and outcomes) at analysis stage according to respective frameworks. The findings on CMO configurations from phase I will inform phases II, III and IV; findings on context and mechanisms will be used to explain outcomes. A realist approach to thematic analysis will be used for this study analysis since it is a suitable method for understanding experiences, thoughts, or behaviours across a data collection [37]. The most frequently accepted framework for conducting thematic analysis is a six-step procedure that includes familiarising oneself with the data, creating initial codes, looking for themes, reviewing themes, defining, and labelling themes, and producing the report [38]. Interpretation and explanation of the qualitative data will be undertaken to allow the refinement and consolidation of context-mechanism-outcome configurations to find out how DFCs work, for people living with dementia, under what circumstances, and how. The synthesis of this data will develop the understanding of how people living with dementia can flourish, or be challenged, in dementia-friendly communities, how informal carers and family members perceive the benefits and challenges of dementia-friendly communities for their loved ones and how staff working in dementia friendly communities perceive their role.

##### Data management

Stage 1, phase I (Realist Review [39]) data will be managed using COVIDENCE software a primary screening tool. Data analysis will use a realist logic of analysis to make sense of the initial programme theory. RAMESES I reporting standards for realist evaluations [20] will be used to identify the evidence base, theory, and potential outcomes. Data to inform the interpretation of the relationships between contexts, mechanisms and outcomes will be sought across documents. Ultimately, analysis will identify context mechanisms influencing how dementia-friendly communities work and showcase the initial theory of how people think they work, which if adopted, may be able to change existing contexts so that ‘key’ mechanisms are triggered to produce desired outcomes.

Stage 2, Phase II data will be collected via field notes alone. Stage 2, Phase III data will be audio-recorded, and Stage 2, phase IV data will be video, and audio recorded. Data will be transcribed verbatim and deidentified by the researcher; thus, protecting confidentiality [40] and uploaded to NVivo11 data management software. A realist approach to thematic analysis will be used for this study analysis since it is a suitable method for understanding experiences, thoughts, or behaviours across a data collection [37]. The most frequently accepted framework for conducting thematic analysis is a six-step procedure that includes familiarising oneself with the data, creating initial codes, looking for themes, reviewing themes, defining, and labelling themes, and producing the report [36]. Interpretation and explanation of the qualitative data will be undertaken allowing us to refine and consolidate our context-mechanism-outcome configurations to find out how DFCs work, for people living with dementia, under what circumstances, and how. The synthesis of this data will develop our understanding about how people living with dementia can flourish, or be challenged, in dementia-friendly communities, how informal carers and family members perceive the benefits and challenges of dementia-friendly communities for their loved ones and how staff working in dementia friendly communities perceive their role. Data will be synthesised using NVivo to code themes through thematic analysis using nodes to identify CMOs. The research team will engage in critical discussions to continue synthesising the results.

### Stage 4

#### Refinement of IPT (Data Synthesis)

The development of context-mechanism-outcome (CMO) configurations will proceed in three broad stages beginning with a realist review of the literature, refined through data collection and subsequent data analysis [41]. The qualitative data will provide in-depth information to refine the CMO configurations from the perspective of those living and working in DFCs. The refinement of proposed CMO configurations will be concluded at this final stage of this study after ongoing refinement at previous stages. Based on the results of all phases, patterns will be analysed, and initial propositions examined and refined. Following this final stage the data will be refined into a programme theory that can be used to help communities in the future. This refined programme theory will help DFCs to be designed, implemented, evaluated and sustained.

##### Setting

The research setting will be active DFCs in NI and data collection will be carried out in various organisations and businesses which will be based on the participants and their community area. These services will advertise themselves as Dementia Friendly through the display of a window decal which is provided by the Alzheimer’s Society following successful completion of their DFC workshops delivered by DFC Champions. As consulted by the Alzheimer’s Society it is not always possible for all staff to receive such training, however, the business or organisation will have been involved in such training to have the accredited decal displayed. In this study, we are observing the experience the person living with dementia has in these settings, not the staff’s dementia friendliness. All observation locations will be in a public setting. As this is an emergent approach the area of observation will be decided in conjunction with the participant keeping in line with the Alzheimer’s Society’s recommended areas (i.e., those organisations that are dementia friendly accredited and/or part of an accredited dementia-friendly community). The settings will all take place within dementia-friendly communities. The Alzheimer’s Society have agreed to act as gatekeepers to support the identification of DFCs.

The Alzheimer’s Society [42] has provided guidance in the UK about the different elements of a community. Consideration of all these areas will support communities to become dementia friendly. The elements are noted in Table 1.

**Table 1:** Example of possible locations for non-participant observation

Observation Numbers	8 Cluster’s recommended by the Alzheimer’s Society	Examples of possible locations for non-participant observation.
**1**	Arts, culture, leisure, and recreation	Theatres
**2**	Businesses and shops	Supermarkets or local businesses
**3**	Children, young people, and students	Charity groups
**4**	Community, voluntary, faith groups and organisations	Community areas
**5**	Emergency Services	Emergency Services
**6**	Health and Social Care	Not Appropriate for non-participant observation as focusing on communal life
**7**	Housing	Housing Associations in Northern Ireland
**8**	Transport	Public transport

## Recruitment

### Consent process

Consent to participate will be gathered from all participants (people with dementia, carers, and staff/volunteers working in a DFC). All participants will be informed that their participation in the study is entirely voluntary. The sample participants will be people with dementia who are independent and living well with dementia, they are individuals with the capacity to make everyday decisions including the ability to manage their own finances [43]. Capacity to consent from people with dementia will be assessed by the gatekeepers within the dementia charities as stipulated by the charity's policies to ensure the safety of their members. The charities involved will share information about the study with members during local group meetings where participant information sheets will be provided. Participants will only be referred to the research team if they have capacity and the gatekeeper is best placed to decide this as they will be familiar with the participant. When participants contact the research team the consent process will be guided by Dewing [44] and consent will be re-checked at the beginning, and conclusion of each part of data collection. Data collection can be terminated at any time.

Process consent will be used to address consent capacity [44]. There are five stages to this process; (1) background and preparation; (2) establishing the basis for capacity; (3) initial consent; (4) ongoing consent monitoring; and (5) feedback and support.

Process consent has been used with the same charity partners in previous studies with researchers in this team [43, 45, 46]. The research team will assess all participants for inclusion and exclusion criteria as stipulated below.

#### Recruitment of people living with dementia

We will conveniently recruit 8-10 people living with dementia who are part of one of two charity organisations in NI for people living with dementia. This number of participants will begin initial theory testing from the literature collected in stage 1. This will allow the research team to evaluate theory from different perspectives compared to typical data collection where saturation is aimed to exhaust participant experience. Recruitment will be facilitated by a gatekeeper in each organisation both gatekeepers have significant experience in this role. Both gatekeepers engage with people with dementia every week in their job and because part of their role is 'empowering people with dementia to contribute to research'. To continue the immersive process of data collection (observational and interviewing), 8-10 people with dementia (with or without family members/ carers) will be recruited for phase II. Semi-structured interviews will take place in phase III with the same identified 8-10 participants recruited in phase II (with or without family members/ carers). For example, this will provide rich data as the researcher will observe what has happened, document data via field notes and explored further with the person with dementia to explore if they have experienced it the same way.

#### Inclusion/ exclusion criteria for people living with dementia

Inclusion criteria

- A dementia diagnosis.
- Person can speak sufficient English to make themselves understood using everyday language.
- Participants should have the capacity to provide process consent for participation at the beginning and throughout the study.
- Participants must be at least 18 years old.

Exclusion criteria

- Participants deemed not to have capacity by gatekeeper.

### Recruitment of relative/ carer

During the initial contact, all potential participants will be asked whether they would like a relative or carer to be with them during data collection (either or both the observation and interview). If so, contact details for the identified relative or carer will be requested by the researcher who will follow up independently with the relative or carer. The relative or carer will follow the same consent process as outlined above (e.g., provide information to the person, provide a cooling off period, the contact person to confirm consent, and meet relative in person (ideally with the person living with dementia) to sign the consent form. No relative/carer will be observed or interviewed without gaining written consent.

#### Inclusion/ exclusion criteria for Family/ carers of people with dementia in a DFC

Inclusion criteria

- Person can speak sufficient English to make themselves understood using everyday language.
- Participants have the capacity to provide consent
- People with experience of supporting someone with dementia in a DFC
- Participants must be at least 18 years old.

Exclusion criteria

- Participants deemed not to have capacity.

### Recruitment of people working/ volunteering in DFCs

Participants working or volunteering in a DFC will be purposively sampled by Alzheimer’s Society who manage a database of all DFCs in NI along with key contact information. After the final observations and participant interviews, the focus group interview will take place (approx. 3 months later). People who are interested in participating in the focus group will choose to self-enrol. Details on how to register, along with eligibility requirements, will be detailed in an information sheet. Following self-enrolment, the researcher will contact each potential participant to confirm their agreement verbally, provide an opportunity to ask questions about the study and complete an online consent form. Due to the wide geographical nature of participants (i.e., across all six counties of NI), these discussions will take place online via MS Teams.

#### Inclusion/ exclusion criteria for Workers/ Volunteers within a DFC

Inclusion criteria

- Person can speak sufficient English to make themselves understood using everyday language.
- Participants have the capacity to provide consent
- Participants must be at least 18 years old.

People who work/ volunteer in DFCs

- Working/ volunteering within an organisation which has received dementia friends training and provides training to employees
- Working in a business displaying ‘Dementia Friendly’ window decal

Exclusion criteria

- Participants deemed not to have capacity.

Institutional consent or consent from the organisations/ businesses involved in this study is not required. This is not required as the research is taking place in a public setting and the focus is on the person living with dementia. As per The British Psychological Society Code of Human Research Ethics [47], only in public settings where people being observed would expect to be observed by strangers is observational study permitted.

### Ethical and governance issues

This study received ethical approval from Faculty Research Ethics Committee MHLS, Queen’s University Belfast in July 2022. (Reference: MHLS 22_78).

Participant fatigue will be monitored throughout all data collection events. A pilot observation and interview will be conducted with a person living with dementia from our ERG before data collection. Following pilot, the experience will be explored with the person living with dementia and the research team who will support each other throughout this study.

Time will be spent participating, and some interviews may touch on challenging or distressing topics. Every participant's participation in the study is completely voluntary, and they are free to leave at any time without needing to give a reason. We do, however, believe that participating in this study will have several advantages. People with dementia, their caregivers or family members, and those who work or volunteer within a DFC are projected to have increased understanding of DFCs. Participation in the study will remain private. Only the research team will have access to participant information. A special identification number will be used to code personal data (a number linked to participants' name which only the research team will have access to on an encrypted file). The identities of participants will be removed from any publications or other outputs, and all information will be kept confidential and secure on Microsoft Teams (UK GDPR compliant). Once the transcript has been written down, digital recordings will be erased. Every piece of information will be handled with absolute confidentiality and in compliance with the 2018 General Data Protection Act [48].

### Rigour

Openness, relevance to practise, commitment to technique, and thoroughness and transparency of data collection/analysis are all linked with rigour in qualitative research [42]. Burns 2009) To guide qualitative research rigour, Lincoln, and Guba [49] present four criteria: credibility, transferability, dependability, and confirmability. Reflective questioning will be employed in all phases of data collection to ensure the study's credibility. Throughout the interviews and focus groups, the research team will consider how they interpreted the participants' responses to fully understand the meaning they were attempting to convey. A selection of participants will review interview transcripts to validate or refute the data as a way of confirming ‘truth’ in the research findings [50] and this will enhance credibility. The transferability of the study will be aided by the researcher’s detailed description of each DFC observed [51]. This will include giving specific explanations of the participants' experiences and going beyond providing surface interpretations to explain the significance of their thoughts, feelings, and behaviours. The study's dependability and confirmability will be established by keeping meticulous records.

### Dissemination

The study is expected to produce several outputs, including conference presentations, webinars, open-access articles in academic journals, and digital media aimed at different stakeholders (such as policymakers, family members, empowerment groups, researchers, and academics). Plans for dissemination will be finalised after consultation between the study team and ERG.

## Discussion

This study outlines the research approach for investigating the experiences of people living with dementia residing in DFCS. The main intention is that through raising awareness and facilitating the development of programme theory the study's conclusions could be applied internationally to help other local communities learn about CMOs for creating and sustaining DFCs. This is especially important in light of the COVID-19 pandemic's increased separation of people living with dementia from their communities and significant reductions in charity support. However, the pandemic has highlighted the importance of effective leadership among professionals and the public [52]. Which will be a crucial aspect in implementing the programme theory into society. Dementia affects more than 55 million individuals worldwide [2]. With significant gaps in both existing literature and community support. This study aims to advance knowledge and provide evidence for the theory underlying the creation of DFCs to assist in providing support for the rising number of people being diagnosed with dementia. This will be done in cooperation with the expert reference group, which consists of people who work in DFCs, reside there, or live with dementia. It is hoped that this study will lead to a cultural shift in how DFCs are seen by the general public and professionals.

## Supplementary Information

**Additional file  1.** 

## Data Availability

De-identifiable data and study materials will be shared, and requests will be judged on an individual basis.
