# Supplementary material for: Exploring the experiences of people living with dementia in Dementia Friendly Communities (DFCs) in Northern Ireland: a realist evaluation protocol
Source: BMC Geriatr. 2023 Jun 9;23:361. doi: 10.1186/s12877-023-04090-y (PMC10251317; doi:10.1186/s12877-023-04090-y)
Supplement: Supplementary file 1 — Additional file 1. [file 12877_2023_4090_MOESM1_ESM.pdf]

Dementia Friendly Communities

QUEEN'S UNIVERSITY BELFAST

NON- PARTICIPANT SEMI- STRUCTURED OBSERVATION GUIDE

Dementia Friendly Communities model:
Venue:
Location:

External Features of environment

	Yes	No	Non-Applicable	Comments
Is there a Dementia Friendly Communities sticker/plaque placed outside the organisation and is it clearly visible?				

Person's engagement with environment (Inside of building)

Are lighting levels appropriate?				
Are the noise levels managed?				
Is there a dedicated space for people with dementia e.g., quiet room?				
Are the seating arrangements dementia friendly?				
Are there appropriate dementia friendly signs for navigation?				
Toilet facilities clearly signposted? What kind of signs are used?				
Is the flooring appropriate?				
Is the building accessible?				
Is there appropriate safety merchandising				

e.g., hazardous products?				
Is there appropriate information at tills and counters?				
Is there a dementia friendly shopping time?				
Are there dementia friendly queues to limit waiting time if the shop is busy?				

Social Interactions with staff or other people:

Do the staff have clear name badges displayed?			
Did the participant disclose their dementia diagnosis?			
Are staff visible at entrance to help?			

Comments on interactions:

For example:

- *How were they spoken to?*
- *What body language was used?*
- *Did they demonstrate any 'dementia friendly' qualities?*
